# Fluid balance and acute lung injury in children and young adults receiving continuous renal replacement therapy: a report from the Worldwide Exploration of Renal Replacement Outcomes Collaborative in Kidney Disease (WE-ROCK)

**DOI:** 10.1007/s00431-026-07285-8

**Published:** 2026-08-04

**Authors:** Zachary A. Rumlow, Jangdong Seo, Amy Strong, Amy Hanson, Todd Jenkins, Ayse Akcan Arikan, Rashid Alobaidi, Aadil Kakajiwala, Zaccaria Ricci, Rachana Srivastava, Matthew Pinto, Sonia Solomon, David T. Selewski, Michelle C. Starr, Shina Menon, Katja M. Gist, Stephen M. Gorga

**Affiliations:** 1 Department of Pediatrics, Division of Critical Care Medicine, University of Michigan Medical School, 1500 E. Medical Center Drive, Ann Arbor, MI F-689048109 USA; 2 Susan B. Meister Child Health Evaluation and Research (CHEAR) Center, University of Michigan, Ann Arbor, MI USA; 3 Department of Biostatistics and Epidemiology, Cincinnati Children’s Hospital Medical Center, Heart Institute Research Core, University of Cincinnati, Ohio, OH USA; 4 Department of Pediatrics, Division of Pediatric Nephrology, Medical School, University of Iowa, Iowa City, IA USA; 5 Department of Pediatrics, Division of Critical Care Medicine, School of Medicine and Public Health, University of Wisconsin, UW Health Kids, Madison, WI USA; 6 Department of Pediatrics, Divisions of Critical Care Medicine and Nephrology, Baylor College of Medicine, Texas Children’s Hospital, Houston, TX USA; 7 Department of Pediatrics, University of Alberta, Edmonton, AB Canada; 8 Department of Pediatrics, Division of Critical Care Medicine, Children’s National Hospital, Washington, DC USA; 9 Department of Emergency and Critical Care, Meyer Children’s Hospital, IRCCS, Florence, Italy; 10 Department of Pediatric, University of California – Los Angeles, Mattel Children’s Hospital, Los Angeles, CA USA; 11 Department of Pediatrics, Westchester Medical Center, Maria Fareri Children’s Hospital, Valhalla, NY USA; 12 Department of Pediatrics, Nemours Children’s Hospital, Orlando, FL USA; 13 Department of Pediatrics, University of Arkansas for Medical Sciences, Little Rock, AR USA; 14 Department of Pediatrics, Indiana University School of Medicine, Riley Children’s Hospital, Indianapolis, IN USA; 15 Department of Pediatrics, Stanford University School of Medicine, Palo Alto, CA USA; 16 Department of Pediatrics, School of Medicine, University of Colorado Anschutz Medical Campus, Children’s Hospital Colorado, Aurora, CO USA

**Keywords:** Continuous renal replacement therapy, Fluid accumulation, Acute respiratory failure

## Abstract

The interactions of pulmonary and renal physiology that underlie acute lung injury (ALI) and acute kidney injury (AKI) have been recognized for nearly a century. The understanding of lung-kidney crosstalk has evolved and is recognized as an important factor in patient management and outcomes. We aim to describe the association between fluid accumulation (FA) and ALI with outcomes of critically ill children and young adults requiring continuous renal replacement therapy (CRRT). Planned secondary analysis using data from the Worldwide Exploration of Renal Replacement Outcomes Collaborative in Kidney Disease (WE-ROCK). ALI severity was defined using the Berlin oxygenation criteria. Fluid accumulation was categorized as ≤ 10%, > 10–20%, and > 20% at CRRT initiation. Illness severity was quantified using the Pediatric Logistic Organ Dysfunction Score 2 (PELOD-2). The primary outcome was intensive care unit (ICU) mortality. Secondary outcomes included (1) 28-day mechanical ventilation (IMV) free days, (2) 28-day ICU free days, and (3) major adverse kidney events at 90 days (MAKE90) defined by death, persistent kidney dysfunction (eGFR reduction by 25% of baseline), or new dialysis requirement. This is a multinational retrospective cohort study. Invasively ventilated patients aged 0–25 years from January 2015 to December 2021 requiring CRRT for AKI or FA with ALI. There were no interventions. A total of 312 patients were included in the analysis. ALI was mild in 67 (21.5%), moderate in 142 (45.5%), and severe in 103 (33.0%). Fluid accumulation was ≤ 10% in 166 (53.2%), > 10–20% in 60 (19.2%), and > 20% in 86 (27.6%). The ICU mortality was 44.6% (139/312). In the multivariable analysis, neither ALI nor FA category was associated with mortality or MAKE90. Time to CRRT initiation (aOR 1.04, 95% CI 1.01–1.08) and illness severity (aOR 1.25, 95% CI 1.14–1.37) were associated with mortality.

*Conclusions*: In children and young adults with ALI receiving CRRT for AKI or FA, longer time to CRRT initiation and illness severity at CRRT initiation were associated with mortality. When accounting for multiple factors, neither ALI nor FA at CRRT initiation were associated with MAKE90 or mortality. Our findings warrant further exploration in a prospective cohort.

**Table:** 

**What is Known:** • *Acute kidney injury (AKI) and acute lung injury (ALI) commonly coexist in critically ill children and are associated with increased morbidity and mortality through bidirectional organ crosstalk.* • *Fluid accumulation (FA) worsens both AKI and ALI, with higher degrees of FA associated with worse outcomes, with continuous renal replacement therapy (CRRT) frequently used to manage the consequences of AKI and FA.*
**What is New:** • *In this multicenter international cohort of children and young adults with ALI receiving CRRT, longer time to CRRT initiation and illness severity at CRRT initiation, but neither ALI nor FA at CRRT initiation were associated with mortality were associated with mortality.* • *These findings suggest that FA thresholds at the start of interventions may be less discriminatory in populations with dynamic processes such as severe multiple organ failure.*

**Supplementary Information:**

The online version contains supplementary material available at 10.1007/s00431-026-07285-8.

## Introduction

Acute kidney injury (AKI) affects 1 in 4 critically ill children and can adversely impact lung function through multiple pathways, including alterations in acid–base balance and fluid accumulation (FA). Acute lung injury (ALI) is a common cause of mortality and morbidity in critical illness, and AKI with or without accompanying FA compounds pulmonary morbidity in children [1–7]. Organ crosstalk is bidirectional, and ALI can affect the kidney by disrupting cell signaling, introducing oxidative stress, and altering hemodynamics, all of which are exacerbated in mechanically ventilated patients [8–10]. It is not surprising that AKI is reported in 1 in 4 patients with Acute Respiratory Distress Syndrome (ARDS) [11–13]. Despite known associations and their common concurrence, specific recommendations regarding the simultaneous management of these pathologies are limited, and practice can vary in management approaches [14].

FA exacerbates ALI and AKI individually and synergistically, and thus international guidelines recommend preventing FA in patients with ALI [15–19]. Indeed, patients with ALI have improved outcomes when they maintain euvolemia [13, 16, 20 ]. Unfortunately, AKI greatly increases the risk for pathologic FA [16, 18, 19, 21, 22Continuous renal replacement therapy (CRRT) is often used to manage fluidbalance in patients with ALI, AKI, and harmful FA [23, 24, 25]. Data on the use of CRRT among patientswith ALI to restore euvolemia have been equivocal and potentially demonstrate that previous work doesnot capture modifiable variables that meaningfully impact patient outcomes [26, 27, 28, 29].

The aim of this study was to examine the association between ALI and FA with intensive care unit (ICU) mortality, invasive mechanical ventilation (IMV) and intensive care unit (ICU) free days, and major adverse kidney events at 90 days (MAKE90) in children and young adults with ALI who received CRRT. We hypothesized that increasing severity of both ALI and FA at CRRT initiation would be associated with worse outcomes.

### Methods

#### Cohort

This was a secondary analysis of data collected for the Worldwide Exploration of Renal Replacement Outcomes Collaborative in Kidney Disease (WE-ROCK) study [30]. WE-ROCK is a retrospective multicenter international study of children and young adults, aged 0–25 years, who received CRRT for AKI or FA from 2015 to 2021. Patients were excluded if they had end-stage renal disease (ESRD), received concurrent extracorporeal membrane oxygenation, peritoneal dialysis before CRRT, or received CRRT for a different indication (for example, liver failure, ingestions and/or inborn errors of metabolism). Data were collected from 35 centers across 9 countries. Each center obtained institutional review board approval, and informed consent was waived due to the study’s retrospective nature. This study follows the Strengthening the Reporting of Observational Studies in Epidemiology (STROBE) guidelines [31].

For this analysis, subjects were included if they had ALI, defined as receipt of IMV and impaired oxygenation at the time of CRRT initiation. Impaired oxygenation was defined by a PF ratio (ratio of the partial pressure of oxygen in arterial blood (PaO2) to the fraction of inspiratory oxygen concentration (FiO2)) of ≤ 300 at CRRT initiation [32]. ALI severity was defined using PF ratio stratification defined in the Berlin definition of ARDS. Accordingly, three ALI categories were defined based on the PF ratio (mild: 200–300, moderate: 100– < 200, severe: ≤ 100) [32]. Patients with cyanotic congenital heart disease, left-ventricular failure, chronic lung disease as a comorbid condition, and/or missing PF ratios were excluded. Although preferred, pediatric acute respiratory distress syndrome (pARDS) could not be identified in this population because (1) chest radiography to identify opacities and mean airway pressure data to calculate oxygenation index were not available and (2) the inability to define the onset of ALI within 7 days of an insult [14]. Baseline creatinine was defined as the lowest value in the preceding 3 months if available or back calculated using an estimated glomerular filtration rate of 100 ml/min/1.73m^2^ [33, 34]. Illness severity was quantified using the Pediatric Logistic Organ Dysfunction Score 2 (PELOD-2) in the 24 h before CRRT initiation as previously described [35].

#### Exposure and outcome variables

Percentage FA was calculated from ICU admission to CRRT initiation using the following equation [28, 36, 37]:

$$\%\;\mathrm{Fluid}\;\mathrm{accumulation}=\lbrack(\mathrm{daily}\;\mathrm{weight}-\mathrm{ICU}\;\mathrm{admission}\;\mathrm{weight})/(\mathrm{ICU}\;\mathrm{admission}\;\mathrm{weight})\rbrack\times100$$

FA categories were stratified into ≤ 10%, > 10–20%, and > 20% based on previously reported literature [28, 36].

The primary exposure of interest was ALI severity at CRRT initiation. Race and ethnicity data were collected and reported as markers of potential socioenvironmental factors associated with differences in outcomes [38, 39]. The primary outcome was ICU mortality. Secondary outcomes included: 28-day IMV-free days, 28-day ICU-free days, and MAKE90, defined as death, persistent kidney dysfunction [eGFR reduction by 25% of baseline], or new dialysis requirement [40]. IMV-free and ICU-free days were 0 for patients who died before 28 days. MAKE90 free days were defined as the number of days without achieving any of the MAKE90 endpoints.

#### Statistical analysis

Continuous variables are presented as median with interquartile range (IQR), and categorical variables are reported as frequencies with percentages (%). Kruskal–Wallis rank sum tests and chi-square tests were used to test differences in continuous and categorical variables, respectively. Patients’ demographics, clinical characteristics, and outcomes were compared by FA at CRRT initiation (≤ 10%, > 10–20%, and > 20%) and ALI severity (mild, moderate, and severe). Multivariable logistic regression was performed to test the association of FA and PF ratio (as continuous variables) at CRRT initiation on death at ICU discharge and MAKE90 Free outcomes after adjusting for the following factors: race, PELOD2 pre-CRRT, time to CRRT initiation (days), and interaction of FA at CRRT initiation with PF ratio. For all patients included in the multi-variable regression, missing values were excluded from the analysis. Two-sided *p*-values < 0.05 were considered statistically significant. All statistical analyses were performed using R version 4.5.0 statistical software (R Core Team, 2025).

## Results

### Patient characteristics

**Fig. 1:**
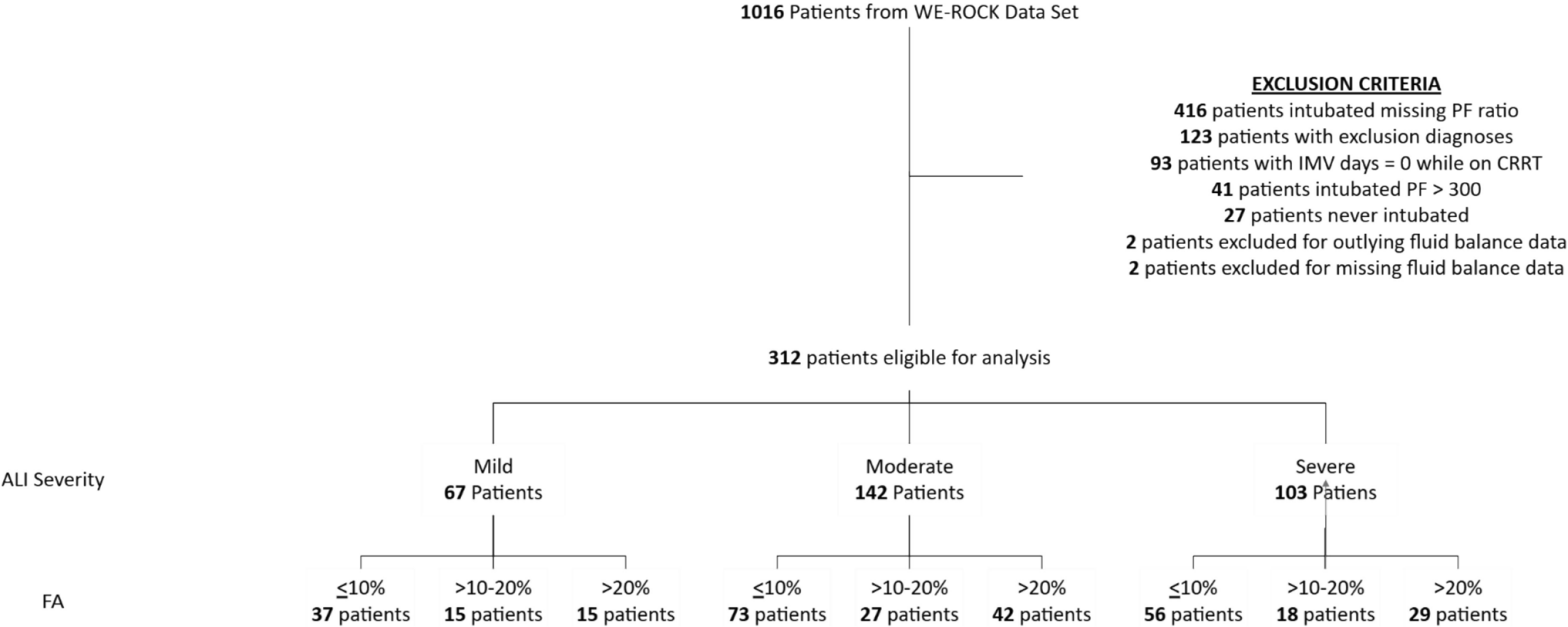
STROBE flow diagram

The WE-ROCK dataset contains 1015 patients who received CRRT. 703 patients were excluded as they did not require invasive mechanical ventilation, did not have an oxygenation defect, had incomplete data, or were extreme outliers, leaving 312 patients eligible for inclusion (Fig. 1). The median age was 7.4 (1.4–14.9) years and 77% had at least one comorbidity. Mild ALI occurred in 67 (21.3%), moderate in 142 (45.5%), and severe in 103 (33.0%) patients (Table 1).

**Table 1:** Demographics, clinical characteristics, and outcomes of patients with ALI stratified by ALI severity

Characteristics	*N*	Overall *N* = 312	Mild ALI 300 ≤ PF < 200 *N* = 67		Moderate ALI 200 ≤ PF < 100 *N* = 142	Severe ALI 100 ≤ PF *N* = 103	*p*-value
**Age**	312	7.4 (1.4, 14.9)	9.6 (1.9, 15.5)	6.1 (1.5, 14.0)		8.0 (1.2, 15.8)	0.45
**Sex**	312						0.65
Female		134 (43%)	32 (48%)	60 (42%)		42 (41%)	
Male		178 (57%)	35 (52%)	83 (58%)		61 (59%)	
**Race**	274						0.02
Asian/Pacific Islander		21 (8%)	6 (10%)	11 (9%)		4 (4%)	
Black		41 (15%)	10 (17%)	12 (10%)		19 (21%)	
More than one race		4 (2%)	0 (0%)	2 (2%)		2 (2%)	
Native Americans		3 (1%)	3 (5%)	0 (0%)		0 (0%)	
White		205 (75%)	39 (67%)	100 (80%)		66 (73%)	
Missing/unknown	38		9	17		12	
**Ethnicity**	275						0.08
Hispanic or Latino		52 (19%)	6 (10%)	29 (23%)		17 (19%)	
Non-Hispanic or Latino		223 (81%)	56 (90%)	96 (77%)		71 (81%)	
Missing/unknown	37		5	17		15	
**Admission category**	312						< 0.001
CNS dysfunction		11 (4%)	6 (9%)	2 (1%)		3 (3%)	
Other		59 (19%)	17 (25%)	29 (20%)		13 (13%)	
Pain/sedation Management		1 (0.3%)	0 (0%)	0 (0%)		1 (1%)	
Post-surgical/minor trauma		14 (5%)	3 (5%)	9 (6%)		2 (2%)	
Respiratory failure		81 (26%)	4 (6.0%)	34 (24%)		43 (42%)	
Shock/infection/major trauma		146 (47%)	37 (55%)	68 (48%)		41 (40%)	
**Sepsis**	312	184 (59%)	42 (63%)	81 (57%)		61 (59%)	0.74
**Comorbidities**	312						0.61
Any		241 (77%)	51 (76%)	107 (75%)		83 (81%)	
None		71 (23%)	16 (24%)	35 (25%)		20 (19%)	
**PaO2/Fio2 ratio**	312	134 (91, 200)	246 (223, 273)	148 (122, 180)		69 (52, 90)	< 0.001
**FA**	312						0.99
< 10%		166 (53%)	37 (55%)	73 (51%)		56 (54%)	
> 10–20%		60 (19%)	15 (22%)	27 (19%)		18 (17%)	
> 20%		86 (28%)	15 (22%)	42 (30%)		29 (28%)	
**PELOD-2 (Pre-CRRT)**	312	8 (6.0, 10.0)	8.0 (5.0, 9.0)	7.0 (5.0, 10.0)		9.0 (7.0, 12.0)	< 0.001
**Pre-CRRT VIS**	311	10 (0, 35)	7 (0, 22)	9.0 (0, 30)		18 (0, 44)	0.08
**Days to CRRT Initiation**	275	2 (1, 6)	2 (1, 4)	3 (1, 7)		2 (1, 7)	0.10

*FA* the percentage fluid balance as a continuous variable at the start of CRRT, *CRRT* continuous renal replacement therapy, *PF Ratio* PaO_2_ to FiO_2_ ratio as a continuous variable at the start of CRRT, *PELOD-2* Pediatric Logistic Organ Dysfunction 2 Score as a continuous variable, *VIS* vasoactive inotropic score, *CI* confidence interval median (Q1, Q3); *n* (%); *MAKE90*, major adverse kidney events at 90 days after acute kidney injury diagnosis

### Fluid accumulation at continuous renal replacement therapy initiation

At CRRT initiation, the FA was ≤ 10% in 166 (53.2%), > 10–20% in 60 (19.2%), and > 20% in 88 (28.2%) patients (Table 2). The distribution of patients between ALI severity and FA is demonstrated in Fig. 1. The ICU mortality was 44.6% (139/312). MAKE90 occurred in 66.3% (207/312) patients, with death in 47.1% (147/312), persistent kidney dysfunction in 15.4% (48/312), and dialysis dependence in 3.8% (12/312) patients, respectively.

**Table 2:** Demographics, clinical characteristics, and outcomes of patients with ALI stratified by FA

Characteristic	*N*	Overall *N* = 312	FA ≤ 10% *N* = 166	10% < FA ≤ 20% *N* = 60	FA > 20% *N* = 86	*p*-value
**Age**	312	7.4 (1.4, 14.9)	12.0 (4.2, 16.1)	5.4 (1.8, 13.0)	2.4 (0.5, 8.0)	< 0.001
**Sex**	312					0.74
Female		134 (43%)	74 (45%)	26 (43%)	34 (40%)	
Male		178 (57%)	92 (55%)	34 (57%)	52 (60%)	
**Race**	274					0.98
Asian/Pacific Islander		21 (7.6%)	13 (8.7%)	3 (5.7%)	5 (7.0%)	
Black		41 (15%)	21 (14%)	8 (15%)	12 (17%)	
More than one race		4 (1.4%)	2 (1.3%)	1 (1.9%)	1 (1.4%)	
Native Americans		3 (1.1%)	3 (2.0%)	0 (0%)	0 (0%)	
White		205 (75%)	111 (74%)	41 (77%)	53 (75%)	
Missing/unknown		38	16	7	15	
**Ethnicity**	275					0.44
Hispanic or Latino		52 (19%)	32 (21%)	7 (14%)	13 (17%)	
Non Hispanic or Latino		223 (81%)	117 (79%)	44 (86%)	62 (83%)	
Missing/unknown	37		17	9	11	
**Admission category**	312					0.01
CNS dysfunction		11 (3.5%)	7 (4.2%)	1 (1.7%)	3 (3.5%)	
Other		59 (19%)	40 (24%)	7 (12%)	12 (14%)	
Pain/sedation management		1 (0.3%)	0 (0%)	1 (1.7%)	0 (0%)	
Post-surgical/minor trauma		14 (4.5%)	5 (3.0%)	4 (6.7%)	5 (5.8%)	
Respiratory failure		81 (26%)	50 (30%)	9 (15%)	22 (26%)	
Shock/infection/major trauma		146 (47%)	64 (39%)	38 (63%)	44 (51%)	
**Sepsis**	312					0.004
No		128 (41%)	82 (49%)	16 (27%)	30 (34%)	
Yes		184 (59%)	84 (51%)	44 (73%)	56 (65%)	
**Comorbidities**	312					0.42
Any		242 (77%)	133 (80%)	45 (75%)	63 (73%)	
None		71 (23%)	33 (20%)	15 (25%)	23 (27%)	
**PaO2/Fio2 ratio**	312	134 (91, 200)	127 (90, 200)	158 (93, 201)	137 (91, 183)	0.45
**ALI severity**	312					0.45
Mild		67 (21%)	37 (22%)	15 (25%)	15 (17%)	
Moderate		142 (46%)	73 (44%)	27 (45%)	42 (49%)	
Severe		103 (33%)	56 (34%)	18 (30%)	29 (34%)	
**PELOD-2 (pre-CRRT)**	312	8 (6.0, 10.0)	8.0 (6.0, 10.0)	9.0 (7.0, 11.0)	8.0 (6.0, 11.0)	0.04
**Pre-CRRT VIS**	311	10 (0, 35)	8 (0, 32)	11 (3, 35)	10 (0, 38)	0.44
**Days to CRRT Initiation**	275	2 (1, 6)	1 (0, 4)	2 (1, 4)	5 (3, 9)	< 0.001

*FA* the percentage fluid balance as a continuous variable at the start of CRRT, *CRRT* continuous renal replacement Therapy, *PF Ratio* PaO_2_ to FiO_2_ ratio as a continuous variable at the start of CRRT, *PELOD-2* Pediatric Logistic Organ Dysfunction 2 Score as a continuous variable, *VIS* vasoactive inotropic score, *CI* confidence interval median (Q1, Q3), *n* (%), *MAKE90* major adverse kidney events at 90 days after acute kidney injury diagnosis

### Characteristics and outcomes stratified by acute lung injury severity

The most common admission category was shock/major infection (47%) (Table 1). Outcomes for patients stratified by ALI severity are described in Table 3. FA categories and time to CRRT initiation were not different between groups. PELOD-2 score was different across the three groups (8 [IQR 5, 9], 7 [IQR 5, 10], 9 [7, 12] *p* < 0.001 for mild, moderate, and severe ALI, respectively). ICU mortality increased across ALI groups, 34% in mild ALI, 40% in moderate ALI and 57% in severe ALI (*p* = 0.005). MAKE90 is described as a three-category variable, with patient assignment to only one outcome (mortality = 147/312, dialysis dependence = 48/312, persistent kidney dysfunction = 12/312). MAKE90 occurred in 74% of patients with severe ALI, but there was no difference between ALI severity groups (*p* = 0.14). Differences in these outcomes were primarily driven by mortality (*p* = 0.01). Patients with severe ALI had fewer ventilator and ICU-free days (*p* = 0.002 and *p* = 0.02, respectively). Patients stratified by ALI showed differences in ALI distribution by race (*p* = 0.02); black patients represented 21% of all patients in the severe ALI category but comprised only 15% of the entire cohort.

**Table 3:** Clinical outcomes of patients with ALI stratified by ALI severity

Characteristic	*N*	Overall *N* = 312	Acute lung injury stratification			*p*-value
			Mild ALI 200 < PF ≤ 300 *N* = 67	Moderate ALI 100 < PF ≤ 200 *N* = 142	Severe ALI PF ≤ 100 *N *= 103	
**CRRT duration**	312	7 (4, 15)	8 (5, 16)	6 (3, 14)	7 (4, 15)	0.27
**28-day VFD**	312	0 (0, 22)	12 (0, 28)	9 (0, 23)	0 (0, 16)	0.002
**28-day ICU free**	312	0.0 (0.0, 5.0)	0.0 (0.0, 6.0)	0.0 (0.0, 8.0)	0.0 (0.0, 0.0)	0.02
**ICU mortality**	312	139 (45%)	23 (34%)	57 (40%)	59 (57%)	0.005
**Hospital Mortality**	312	147 (47%)	25 (37%)	62 (44%)	60 (58%)	0.02
**MAKE-90**	312	207 (66%)	43 (64%)	88 (62%)	76 (74%)	0.14
Death at 90 days		147 (47%)	26 (39%)	82 (58%)	42 (41%)	0.01
Kidney dysfunction		48 (15%)	12 (18%)	23 (16%)	13 (13%)	0.61
Dialysis dependence		12 (4%)	5 (8%)	5 (4%)	2 (2%)	0.23

*FA* the percentage fluid balance as a continuous variable at the start of CRRT, *CRRT* continuous renal replacement therapy, *PF Ratio* PaO_2_ to FiO_2_ ratio as a continuous variable at the start of CRRT, *PELOD-2* Pediatric Logistic Organ Dysfunction 2 Score as a continuous variable, *VFD* ventilator free days, *CI* confidence interval median (Q1, Q3), *n* (%) *MAKE90*, major adverse kidney events at 90 days after acute kidney injury diagnosis

### Characteristics and outcomes stratified by fluid balance

Outcomes for patients stratified by FA are described in Supplemental Table 1. Patients with higher FA were younger (2.4 years [IQR 0.5, 8.0], *p* < 0.001) and more likely to have sepsis at the time of CRRT initiation (*p* = 0.004). Further, greater FA was associated with later initiation of CRRT (1 day [IQR 0, 4], 2 days [IQR 1, 4], 5 days [IQR 2, 10], *p* < 0.001 for ≤ 10%, > 10–20%, > 20%, respectively). ALI severity as measured by PF ratio was similar between fluid strata (Table 2).

There was no association between the FA categories and ICU mortality (48% vs 45% vs 38% for ≤ 10%, > 10–20%, and > 20%, respectively (*p* = 0.38)). There was no association between FA strata and IMV-free days, ICU-free days, or ICU mortality (*p* > 0.05). Finally, there were no associations between FA strata and MAKE90 or any of its components (*p* > 0.05).

### Interaction of fluid accumulation and acute lung injury

To assess the interaction between FA and ALI severity on ICU mortality, we performed multivariable regression analysis including the PF ratio, FA, and an interaction term of PF ratio and FA as continuous variables (Supplemental Tables 2b and 3b). A priori, we evaluated interactions between PF ratio and FA, and between FA and time to CRRT. We present unadjusted sensitivity analyses without the interaction term in Supplemental Tables 2a and 3a. After adjusting for the interaction terms, race, PELOD-2 score, and time to CRRT initiation, neither interaction term demonstrated an association with mortality.

Longer time to CRRT initiation, as a continuous variable (aOR 1.04, 95% CI 1.01–1.08; *p* = 0.01), and PELOD-2 (aOR 1.25, 95% CI 1.14–1.35; *p* ≤ 0.001) were associated with increased odds of ICU mortality. Similarly, both shorter time to CRRT initiation (aOR 0.95, 95% CI 0.91–0.99; *p* = 0.015) and lower PELOD-2 (aOR 0.86, 95% CI 0.79–0.94; *p* < 0.001) were associated with lower odds of MAKE90 outcomes.

## Discussion

In this secondary analysis of children and young adults receiving CRRT for AKI or FA with ALI on IMV, we found that time to CRRT initiation and higher PELOD-2 scores, neither ALI severity nor FA, were associated with ICU mortality or MAKE90 outcomes. These findings stand in contrast to previous studies of ALI severity and FA, which may be related to the degree and severity of multiple organ failure in our cohort, compared to those in prior landmark literature [5, 36]. Notably, more than 50% of the cohort had %FA < 10% at CRRT initiation across all ALI groups, which contrasts with previously published literature [36].

We demonstrated that ALI severity was associated with ICU mortality on univariate analysis; however, we did not find an association between the degree of oxygenation defect and mortality or MAKE90 after accounting for confounding factors, including FA, race, illness severity, and time to CRRT initiation. Two important reasons are possible. First, the causes of the oxygenation defect were not reported. We could not identify if the driver of the oxygenation defect was related to primary parenchymal disease, secondary parenchymal injury, or interstitial pulmonary edema from the pathologic impact of FA that was present at CRRT initiation. Evidence suggests that the underlying cause of ALI likely has different impacts on mortality [5]. Further, it is plausible that each of these etiologies would respond differently to CRRT and thereby drive unmeasured confounding and bias the overall population towards the null [7, 41]. Second, we found that ICU mortality in this population was measurably higher than in previous analyses. Here, we found that children and young adults with ALI requiring CRRT for AKI or FA had a mortality of 47%. Previous studies, including landmark worldwide epidemiological studies of pARDS, report an overall mortality of 17% [5]. Additional work by Zinter et al. reported a mortality of 29% in a cohort of patients with pARDS and positive fluid balance [13]. Our findings suggest that in a population with severe multiple organ failure, requiring CRRT, the static threshold of fluid accumulation may be less discriminatory for outcomes, which may explain this difference compared to historic studies. Our group exclusively includes patients with multiple organ failures who had an oxygenation defect and AKI or FA requiring at least IMV and CRRT for management. Presence of multiple organ failure as a driver of mortality in a population with pARDS has previously been described by Dowell et al., who demonstrated that the underlying multiple organ failure and not the severity of ALI was the driver of mortality in a pARDS population [42]. Importantly, given the nature of data collection for our analysis, we are unable to specifically comment on the etiologies of pulmonary failure and how they may modify the relationship between AKI and CRRT [41]^***^. Further studies investigating the causes of ALI at CRRT initiation, in particular differentiating patients exclusively with pulmonary edema requiring fluid removal and those with parenchymal diseases with or without pulmonary edema, may help understand this population and the potential therapeutic benefits of fluid removal.

Similarly, we did not find an association with ICU mortality or MAKE90 with the degree of FA. Multiple previous studies have demonstrated an association with positive fluid balance and mortality in many critically ill pediatric populations, including those requiring CRRT [6, 17, 28, 36, 43, 44]. In a multicenter study of patients receiving CRRT, Sutherland et al. demonstrated that a FA greater than 20% was associated with increased mortality [36]. Three major factors may explain this contrast. First, the presence of multiple organ failure itself may blunt the direct impact of fluid balance severity. In Sutherland’s cohort (despite similar fluid balance), mortality was substantially higher in the presence of multiple organ failure compared to those without (OR 4.66 vs 1.03, respectively). Secondly, we cannot account for differences in practice occurring over time. Indeed, practice changes over time may be the driver of lower %FA across the entire cohort, thus diluting the association of FA with outcomes in the larger cohort. Within the larger WE-ROCK study population, CRRT was initiated at a median FA of 7.4%, well below previously identified thresholds. Within the WE-ROCK cohort, each day of delay of CRRT initiation was associated with increased odds of mortality [47]. Given the similarity in outcomes in our population with regard to timing of CRRT initiation, it is possible that the benefit of CRRT is more complex than restoring an appropriate fluid status [41, 42]. The ideas surrounding timing and prescription of fluid removal by CRRT within a patient’s course have been identified as areas for further investigation, as they likely impact patient outcomes [37, 45, 46]. Further identification of patients who would benefit from more nuanced approaches to fluid balance management, as well as understanding fluid balance trajectories during illness and the multifactorial impact of CRRT therapy, may improve understanding of the impact of FA and renal function on patient outcomes.

We showed that after accounting for multiple factors, only shorter time to CRRT initiation and lower PELOD-2 score were associated with reduced odds of MAKE90. In contrast to previous work, our data suggests that FA is not associated with differences in mortality nor MAKE90. These findings reinforce that global illness severity and multiple organ failure may improve prediction of mortality compared to isolated, organ-specific markers of illness. The association with higher PELOD-2 score and worse outcomes is consistent with multiple other studies which have demonstrated that patients with more severe illness and multiple organ failure have worse outcomes than those with less severe illness and single organ failure [47–49]. In particular, the known association between AKI and ALI, and their combined effects on outcomes, is well described [41]. Among the mechanically ventilated population, the neurohormonal activation, hemodynamic alterations, and cellular signaling changes can promote and exacerbate AKI and FA [41, 50]. While positive, our results stand in contrast to previous interventional trials and should be interpreted cautiously [51]. Especially in younger populations, delayed time to CRRT may mediate a relationship between ongoing complex multiple organ failure rather than suggest a protective impact against the harmful effects of ALI and FA [52]. Thusly, the impact of CRRT upon mediation of harmful cellular signaling and neurohormonal remains an area for future research [41].

The strength of this study lies in its large sample size and multicenter nature, increasing the generalizability of our findings. However, we do recognize that there are several limitations. Importantly, for continuous variables such as PF ratio and fluid balance, only single measurements at the time of CRRT initiation exist within the available data. We recognize that both of these variables may represent dynamic changes in lung and renal injury, and as such, we fail to capture potentially important data when considering the implications of our analysis. Further, patient data was captured only at the site of CRRT utilization. Therefore, there were incomplete data surrounding baseline weights and FA. As defined, our cohort includes only patients who received CRRT without a control group and cannot make causal claims. We also recognize the variable practice patterns of individual centers and providers that influence fluid accumulation, and these were not captured. Accordingly, the complete fluid status and response to it by clinicians cannot be fully quantified and may have inappropriately over-influenced non-significant effect of FA on outcomes. Given the nature of how data were collected and reported, we do not report exposures as they relate to CRRT prescription, including the lack of information regarding net ultrafiltration. This variable likely represents a modifiable variable that modifies the impact of fluid accumulation. Given its exclusion, we cannot report the impact of different CRRT prescriptions on patient outcomes. Lastly, we have applied a generalized definition of ALI to our cohort. We recognize that ALI and oxygenation defects are not monolithic in nature and may respond variably to fluid accumulation. Further, we do not report an IMV group without ALI to represent potential differences in response to therapy for patients without an oxygenation defect.

## Conclusions

In this analysis of a large multi-center cohort of children and young adults with ALI who required CRRT for either AKI or fluid accumulation, neither ALI nor FA severity were associated with mortality nor MAKE90 as outcomes. Longer time to CRRT initiation and higher illness severity scores were associated with mortality and MAKE90. In this cohort, the degree of FA is lower than in previous reports, suggesting an impact in changing epidemiology and evolving clinical practice. While limited in our ability to causally describe the impact of CRRT and specific variables on outcomes, we highlight differences from historic data and emphasize that commonly used static measures of fluid accumulation may be insufficient in identifying patients at greatest risk of adverse outcomes. Identifying risk factors and appropriate therapeutic targets and modalities targets to improve salient outcomes may require more refined phenotyping of patients with ALI.

## Supplementary Information

Below is the link to the electronic supplementary material.

ESM 1 (DOCX 49.2 KB)

## Data Availability

Data are housed as Cincinnati Children’s Hospital and Medical Center and are available upon request.

## Declarations

### Artificial intelligence

No artificial intelligence was used during this reporting process, including manuscript preparation and submission.
