# Supplementary material for: Fluid balance and acute lung injury in children and young adults receiving continuous renal replacement therapy: a report from the Worldwide Exploration of Renal Replacement Outcomes Collaborative in Kidney Disease (WE-ROCK)
Source: Eur J Pediatr. 2026 Aug 4;185(8):638. doi: 10.1007/s00431-026-07285-8 (PMC13437644; doi:10.1007/s00431-026-07285-8)
Supplement: Supplementary file 1 [file 431_2026_7285_MOESM1_ESM.docx]

**Supplemental Materials**

Fluid accumulation and Acute Lung Injury in Children and young Adults receiving Continuous Renal Replacement Therapy: A Report From The Worldwide Exploration of Renal Replacement Outcomes Collaborative in Kidney Disease (WE-ROCK)

Zachary A. Rumlow DO^1,2^, Jangdong Seo PhD^3^, Amy Strong MD, MSc^4^, Amy Hanson^5^, Todd Jenkins PhD, MPH^3^, Ayse Akcan Arikan MD^6^, Rashid Alobaidi MD^7^, Aadil Kakajiwala MD MSCI^8^, Zaccaria Ricci MD^9^, Rachana Srivastava MD^10^, Matthew Pinto MD^11^, Sonia Solomon MD^12^, David T. Selewski MD MSCR^13^, Michelle C. Starr MD MPH^14^, Shina Menon MD^15^, Katja M. Gist DO MS^16^, Stephen M. Gorga MD, MSc^1^

Department of Pediatrics, Division of Critical Care Medicine, University of Michigan Medical School, Ann Arbor, Michigan USA^1^

Susan B. Meister Child Health Evaluation and Research (CHEAR) Center, University of Michigan, Ann Arbor, Michigan, USA^2^

Department of Biostatics and Epidemiology, Heart Institute Research Core, Cincinnati Children's Hospital Medical Center, University of Cincinnati, Ohio, USA^3^

Department of Pediatrics, Division of Pediatric Nephrology, University of Iowa Medical School, Iowa City, Iowa USA^4^

Department of Pediatrics, Division of Critical Care Medicine, University of Wisconsin School of Medicine and Public Health, UW Health Kids, Madison, Wisconsin, USA^5^

Department of Pediatrics, Divisions of Critical Care Medicine and Nephrology. Baylor College of Medicine, Texas Children’s Hospital, Houston, Texas, USA^6^

Department of Pediatrics, University of Alberta, Edmonton, Alberta, Canada^7^

Department of Pediatrics, Division of Critical Care Medicine, Children’s National Hospital, Washington DC, USA^8^

Department of Emergency and Critical Care, Meyer Children’s Hospital, IRCCS, Florence, Italy^9^

Department of Pediatrics, University of California – Los Angeles, Mattel Children’s Hospital, Los Angeles, California, USA^10^

Department of Pediatrics, Westchester Medical Center, Maria Fareri Children’s Hospital, Valhalla, New York, USA^11^

Department of Pediatrics, Nemours Children’s Hospital, Orlando, Florida, USA^12^

Department of Pediatrics, University of Arkansas for Medical Sciences, Little Rock, AR, USA^13^

Department of Pediatrics, Indiana University School of Medicine, Riley Children’s Hospital, Indianapolis, Indiana, USA^14^

Department of Pediatrics, Stanford University School of Medicine, Palo Alto, California, USA^15^

Department of Pediatrics, University of Colorado Anschutz Medical Campus, School of Medicine, Children’s Hospital Colorado, Aurora CO, USA^16^

Table of Contents:

Supplemental

Collaborative coauthor list ………………………………………………………………………………………………………………………….………3-5

Acknowledgements ………………………………………………………………………………………………………………………………………………..5

Tables:…………………………………………………………………………………………………………………………………………………………………6-13

Supplemental Table 1…………………………………………………………………………………………………………………………………6

Supplemental Table 2a……………………………………………………………………………………………………………………………….7

Supplemental Table 2b……………………………………………………………………………………………………………………………….8

Supplemental Table 3a……………………………………………………………………………………………………………………………….9

Supplemental Table 3b……………………………………………………………………………………………………………………………..10

Supplemental Table 4……………………………………………………………………………………………………………………………….11

Supplemental Table 5…………………………………………………………………………………………………………………………….…12

Supplemental Table 6…………………………………………………………………………………………………………………………….…13

Supplemental Table 7…………………………………………………………………………………………………………………………….…14

References: :………………………………………………………………………………………………………………………………………………………….15

**WE ROCK Collaborative Author List**

The following individuals served as collaborators and investigators for the WE-ROCK studies. They collaborated in protocol development and review, data analysis, and participated in drafting or review of the manuscript, and their names should be citable by PubMed.

Emily Ahern CPNP, DNP^1^, Ayse Akcan Arikan MD^2^, Issa Alhamoud MD^3^, Rashid Alobaidi MD, MSc^4^, Pilar Anton-Martin MD, PhD^5,6^, Shanthi S Balani MD^7^, Matthew Barhight MD, MS^8^, Abby Basalely MD, MS^9^, Amee M Bigelow MD, MS^10^, Gabriella Bottari MD^11^, Andrea Cappoli MD^11^, Abhishek Chakraborty MD^5^, Eileen A Ciccia MD^12^, Michaela Collins BA^13^, Denise Colosimo MD^14^, Gerard Cortina MD^15^, Mihaela A Damian MD, MPH^16,17^, Sara De la Mata Navazo MD^18^, Gabrielle DeAbreu MD^9^, Akash Deep MD^19^, Kathy L Ding BS^20^, Kristin J Dolan MD^2^, Lama Elbahlawan MD^21^, Sarah N Fernandez Lafever MD, PhD^18^, Dana Y Fuhrman DO, MS^13,22^, Ben Gelbart MBBS^23^, Katja M Gist DO MSc^1,20^, Stephen M Gorga MD, MSc^24^, Francesco Guzzi MD^25^, Isabella Guzzo MD^11^, Taiki Haga MD^26^, Elizabeth Harvey MD^27^, Denise C Hasson MD^28^, Taylor Hill-Horowitz BS^9^, Haleigh Inthavong BS, MS^2^, Catherine Joseph MD^2^, Ahmad Kaddourah MD, MS^29^, Aadil Kakajiwala MD, MSCI^30^, Aaron D Kessel MD, MS^9^, Sarah Korn DO^31^, Kelli A Krallman BSN, MS^13^, David M Kwiatkowski MD Msc^16^, Jasmine Lee MSc^27^, Laurance Lequier MD^4^, Tina Madani Kia BS^4^, Kenneth E Mah MD, MS^16,32^, Eleonora Marinari MD^11^, Susan D Martin MD^33^, Shina Menon MD^16,34^, Tahagod H Mohamed MD^10^, Catherine Morgan MD MSc^4^, Theresa A Mottes APRN^8^, Melissa A Muff-Luett MD^35^, Siva Namachivayam MBBS^23^, Tara M Neumayr MD^12^, Jennifer Nhan MD, MS^7,30^, Abigail O'Rourke MD^9^, Nicholas J Ollberding PhD^13^, Matthew G Pinto MD^36^, Dua Qutob MD^29^, Valeria Raggi MD^11^, Stephanie Reynaud MD^37^, Zaccaria Ricci MD^14^, Zachary A Rumlow DO^3,24^, María J Santiago Lozano MD, PhD^18^, Emily See MBBS^23^, David T Selewski MD, MSCR^38,39^, Carmela Serpe MSc, PhD^11^, Alyssa Serratore RN, MsC^23^, Ananya Shah BS^20^, Weiwen V Shih MD^1,20^, H Stella Shin MD^17^, Cara L Slagle MD^40^, Sonia Solomon DO^36,41^, Danielle E Soranno MD^40^, Rachana Srivastava MD^43^, Natalja L Stanski MD^13^, Michelle C Starr MD, MPH^40^, Erin K Stenson MD^1,20^, Amy E Strong MD, MSCE^3^, Susan A Taylor MSc^19^, Sameer V Thadani MD^2^, Amanda M Uber DO^35,42^, Brynna Van Wyk ARNP, MSN^3^, Tennille N Webb MD, MSPH^44^, Huaiyu Zang PhD^13^, Emily E Zangla DO^7^, Michael Zappitelli MD, MSc^27^

^1^Children's Hospital Colorado, Aurora, CO, USA

^2^Baylor College of Medicine, Texas Children's Hospital, Houston, TX, USA

^3^University of Iowa Stead Family Children's Hospital, Carver College of Medicine, Iowa City, IA, USA

^4^Univeristy of Alberta, Edmonton, Canada

^5^Le Bonheur Children's Hospital, Memphis, TN, USA

^6^Children’s Hospital of Philadelphia, University of Pennsylvania Perelman School of Medicine, PA, USA

^7^University of Minnesota, Minneapolis, MN, USA

^8^Ann and Robert H. Lurie Children's Hospital of Chicago, Chicago, IL, USA

^9^Cohen Children's Medical Center, Zucker School of Medicine, New Hyde Park, NY, USA

^10^Nationwide Children's Hospital, The Ohio State University College of Medicine, Columbus, OH, USA

^11^Bambino Gesù Children's Hospital, IRCCS, Rome, Italy

^12^Washington University School of Medicine, St. Louis Children's Hospital, St. Louis, MO, USA

^13^Cincinnati Children's Hospital Medical Center; University of Cincinnati College of Medicine, Cincinnati, OH, USA

^14^Meyer Children's Hospital, IRCCS, Florence, Italy

^15^Medical University of Innsbruck, Innsbruck, Austria

^16^Stanford University School of Medicine, Palo Alto, CA, USA

^17^Children's Healthcare of Atlanta, Emory University School of Medicine, Atlanta, GA, USA

^18^Gregorio Marañón University Hospital; School of Medicine, Madrid, Spain

^19^King's College Hospital, London, England

^20^University of Colorado, School of Medicine, Aurora, CO, USA

^21^St Jude Children’s Hospital, Memphis TN USA

^22^University of Pittsburgh Medical Center Children's Hospital of Pittsburgh, Pittsburgh, PA, USA

^23^Royal Children's Hospital, University of Melbourne, Murdoch Children's Research Institute, Melbourne, Victoria, Australia

^24^University of Michigan Medical School, C.S. Mott Children's Hospital, Ann Arbor, MI, USA

^25^Santo Stefano Hospital, Prato, Italy

^26^ Osaka City General Hospital, Osaka, Japan

^27^Hospital for Sick Children, Toronto, Ontario, Canada

^28^NYU Langone Health, Hassenfeld Children’s Hospital, New York, NY, USA

^29^ Sidra Medicine and Weil Cornel Medicine, Qatar, Doha, Qatar

^30^Children's National Hospital, Washington, DC, USA

^31^Westchester Medical Center, Westchester, NY, USA

^32^University of California, San Francisco, CA, USA

^33^Golisano Children's Hospital at University of Rochester Medical Center, Rochester, NY, USA

^34^Seattle Children's Hospital, University of Washington, Seattle, WA, USA

^35^University of Nebraska Medical Center, Children's Hospital & Medical Center, Omaha, NE, USA

^36^Department of Pediatrics, Westchester Medical center, Maria Fareri Children's, Valhalla, NY, USA

^37^Dalhouse University, Halifax, Nova Scotia, Canada

^38^Medical University of South Carolina, Charleston, SC, USA

^39^Arkansas Children’s Hospital, University of Arkansas for Medical Sciences, Little Rock, AR, USA

^40^Indiana University School of Medicine, Riley Hospital for Children, Indianapolis, IN, USA

^41^Department of Pediatrics, Nemours Children’s Hospital, Wilmington DE, USA

^42^University of Utah School of Medicine, Primary Children’s Hospital, Salt Lake City, UU, USA

^43^Mattel Children's Hospital at UCLA, Los Angeles, Ca, USA

^44^Children's of Alabama/University of Alabama at Birmingham, Birmingham, AL, USA,

**Acknowledgements**

T. Christine E. Alvarez MHI RN^1^, Elizabeth Bixler BS^2^, Erica Blender Brown MA, CRA^3^, Cheryl L Brown BS^1^, Ambra Burrell BA^4^, Anwesh Dash BS^5^, Jennifer L Ehrlich RN MHA^6^, Simrandeep Farma HBSc^7^, Kim Gahring RN BSN, CCRN^8^, Barbara Gales RN^9^, Madison R Hilgenkamp^10^, Sonal Jain MS^11^, Kate Kanwar BA MS^4^, Jennifer Lusk BSN RN, CCRN^8^, Christopher J. Meyer BA AA^1^, Katherine Plomaritas BSN RN^12^, Joshua Porter BS^5^, Jessica Potts BSN RN^13^, Alyssa Serratore BNurs, GDipNP(PIC), RN, MsC^14^, Elizabeth Schneider BS^5^, Vidushi Sinha BS^5^, PJ Strack RN,BSN,CCRN^15^, Sue Taylor RN^16^, Katherine Twombley MD^3^, Brynna Van Wyk MSN, ARNP CPNP^6^, Samantha Wallace MS^17^, Janet Wang BS^5^, Megan Woods BS^5^, Marcia Zinger RN^18^, Alison Zong BS^5^

^1^Cincinnati Children's Hospital Medical Center, Cincinnati, OH, USA

^2^Baylor College of Medicine, Texas Children's Hospital, Houston, TX, USA

^3^Medical University of South Carolina, Charleston, SC, USA

^4^Nationwide Children's Hospital, Columbus, OH, USA

^5^University of Tennessee Health Science Center College of Medicine, Memphis, TN, USA

^6^University of Iowa Stead Family Children's Hospital, Carver College of Medicine, Iowa City, IA, USA

^7^Hospital for Sick Children, Toronto, ON, Canada

^8^Children's Hospital Colorado, Aurora, CO, USA

^9^Mattel Children’s Hospital at UCLA, Los Angeles, CA, USA

^10^University of Nebraska Medical Center, Children's Hospital & Medical Center, Omaha, NE, USA

^11^Seattle Children's Hospital, Seattle, WA, USA

^12^University of Michigan, C.S. Mott Children's Hospital, Ann Arbor, MI, USA

^13^Children's of Alabama/University of Alabama at Birmingham, Birmingham, AL, USA

^14^Royal Children's Hospital, Melbourne, VIC, Australia

^15^Children's Mercy Hospital, Kansas City, MO, USA

^16^King's College Hospital, London, England

^17^Indiana University School of Medicine, Riley Hospital for Children, Indianapolis, IN, USA

^18^Cohen Children's Medical Center, New Hyde Park, NY, USA

Supplemental Table 1. Univariate Stratification of Outcome for Patients stratified by Fluid accumulation strata

| **Fluid accumulation Stratification** | |  |  |  |  |  |
| --- | --- | --- | --- | --- | --- | --- |
| **Characteristic** | **N** | **Overall**  **N = 312** | **≤10%**,  N = 166 | **>10-20%**,  N = 60 | **>20%**,  N = 86 | **p-value** |
| **CRRT duration** | 312 | 7 (4, 15) | 7 (3, 15) | 7 (4, 15) | 6 (4, 14) | 0.99 |
| **28-day VFD** | 312 | 0 (0, 22) | 0 (0, 22) | 0 (0, 20) | 5 (0, 28) | 0.45 |
| **28-day ICU free** | 312 | 0 (0, 5) | 0 (0, 6) | 0 (0, 6.5) | 0(0, 0) | 0.16 |
| **ICU Mortality** | 312 | 139 (45%) | 79 (48%) | 27 (45%) | 33 (38%) | 0.38 |
| **Hospital Mortality** | 312 | 147 (47%) | 84 (51%) | 29 (48%) | 34 (40%) | 0.24 |
| **MAKE-90** | 312 | 207 (66%) | 117 (70%) | 39 (65%) | 51 (59%) | 0.20 |
| Death | 312 | 147 (47%) | 83 (50%) | 29 (48%) | 35 (41%) | 0.37 |
| Kidney dysfunction | 312 | 48 (15%) | 27 (16%) | 10 (17%) | 11 (13%) | 0.73 |
| Dialysis Dependence | 312 | 12 (4%) | 7 (4%) | 0 (0%) | 5 (6%) | 0.16 |

Median (Q1, Q3); n (%)

### Abbreviations: FA^1^ –the percentage fluid accumulation as a continuous variable at the start of CRRT; CRRT – Continuous Renal Replacement Therapy; PF Ratio^2^ – PaO_2_ to FiO_2_ ratio as a continuous variable at the start of CRRT OR – Odds Ratio; MAKE90^3^ – Major Adverse Kidney Events at 90 days after acute kidney injury diagnosis; PELOD-2^4^ – Pediatric Logistic Organ Dysfunction 2 Score as a continuous variable; Time to CRRT – time in days from intensive care admission to CRRT initiation; CI – Confidence Interval Median (Q1, Q3); n (%);

### Supplemental Table 2a. Multivariable assessment of Fluid Accumulation and PF ratio on death in the ICU, without Interaction term

| **Characteristic** | **OR** | **95% CI** | **p-value** |
| --- | --- | --- | --- |
| FA at CRRT Initiation | 0.96 | 0.76 – 1.20 | 0.724 |
| PF Ratio at CRRT Initiation | 0.66 | 0.52 – 0.84 | <0.001 |

### Abbreviations: FA^1^ –the percentage fluid accumulation as a continuous variable at the start of CRRT; CRRT – Continuous Renal Replacement Therapy; PF Ratio^2^ – PaO_2_ to FiO_2_ ratio as a continuous variable at the start of CRRT OR – Odds Ratio; MAKE90^3^ – Major Adverse Kidney Events at 90 days after acute kidney injury diagnosis; PELOD-2^4^ – Pediatric Logistic Organ Dysfunction 2 Score as a continuous variable; Time to CRRT – time in days from intensive care admission to CRRT initiation; CI – Confidence Interval Median (Q1, Q3); n (%);

Supplemental Table 2b. Multivariate assessment of the interaction of Fluid Accumulation and PF ratio on death in the ICU, with Interaction term

| **Characteristic** | **OR** | **95% CI** | | **p-value** |
| --- | --- | --- | --- | --- |
| FA at CRRT Initiation | 0.79 | 0.57 – 1.05 | | 0.13 |
| PF Ratio at CRRT Initiation | 0.83 | 0.62 – 1.10 | | 0.194 |
| Race |  |  |  |  |
| Asian/Pacific Islander | - | - | | - |
| Black | 0.45 | 0.14 – 1.45 | | 0.18 |
| More than one race | 0.70 | 0.06 – 8.12 | | 0.77 |
| Native American | 0.00 | - | | 0.99 |
| White | 0.58 | 0.21 – 1.58 | | 0.28 |
| Pre-CRRT PELOD-2 | 1.25 | 1.14 – 1.35 | | < 0.001 |
| Time to CRRT initiation (days) | 1.04 | 1.01 – 1.08 | | 0.01 |
| FA and PF Ratio Interaction | 1.03 | 0.70 – 1.44 | | 0.88 |

### Abbreviations: FA^1^ –the percentage fluid accumulation as a continuous variable at the start of CRRT; CRRT – Continuous Renal Replacement Therapy; PF Ratio^2^ – PaO_2_ to FiO_2_ ratio as a continuous variable at the start of CRRT OR – Odds Ratio; MAKE90^3^ – Major Adverse Kidney Events at 90 days after acute kidney injury diagnosis; PELOD-2^4^ – Pediatric Logistic Organ Dysfunction 2 Score as a continuous variable; Time to CRRT – time in days from intensive care admission to CRRT initiation; CI – Confidence Interval Median (Q1, Q3); n (%);

### Supplemental Table 2a. Multivariable assessment of Fluid Accumulation and PF ratio on MAKE-90, without Interaction term

| **Characteristic** | **OR** | **95% CI** | **p-value** |
| --- | --- | --- | --- |
| FA at CRRT Initiation | 1.08 | 0.86 – 1.37 | 0.493 |
| PF Ratio at CRRT Initiation | 1.35 | 1.06 – 1.71 | 0.015 |

### Abbreviations: FA^1^ –the percentage fluid accumulation as a continuous variable at the start of CRRT; CRRT – Continuous Renal Replacement Therapy; PF Ratio^2^ – PaO_2_ to FiO_2_ ratio as a continuous variable at the start of CRRT OR – Odds Ratio; MAKE90^3^ – Major Adverse Kidney Events at 90 days after acute kidney injury diagnosis; PELOD-2^4^ – Pediatric Logistic Organ Dysfunction 2 Score as a continuous variable; Time to CRRT – time in days from intensive care admission to CRRT initiation; CI – Confidence Interval Median (Q1, Q3); n (%);

Supplemental Table 3b. Multivariate assessment of the interaction of Fluid Accumulation and PF ratio on MAKE90 with Interaction term

| **Characteristic** | **OR** | **95% CI** | | **p-value** |
| --- | --- | --- | --- | --- |
| FA at CRRT Initiation | 1.25 | 0.94 – 1.69 | | 0.13 |
| PF Ratio at CRRT Initiation | 1.12 | 0.84 – 1.49 | | 0.45 |
| Race |  |  |  |  |
| Asian/Pacific Islander | - | - | | - |
| Black | 1.41 | 0.45 – 4.57 | | 0.56 |
| More than one race | 0.61 | 0.03 – 6.67 | | 0.71 |
| Native American | 0.71 | 0.03 – 8.98 | | 0.80 |
| White | 0.88 | 0.33 – 2.42 | | 0.79 |
| Pre-CRRT PELOD-2 | 0.86 | 0.79 – 0.94 | | < 0.001 |
| Time to CRRT Initiation (days) | 0.95 | 0.91 – 0.99 | | 0.015 |
| FA and PF Ratio Interaction | 0.99 | 0.72 – 1.40 | | 0.96 |

### Abbreviations: FA^1^ –the percentage fluid accumulation as a continuous variable at the start of CRRT; CRRT – Continuous Renal Replacement Therapy; PF Ratio^2^ – PaO_2_ to FiO_2_ ratio as a continuous variable at the start of CRRT OR – Odds Ratio; MAKE90^3^ – Major Adverse Kidney Events at 90 days after acute kidney injury diagnosis; PELOD-2^4^ – Pediatric Logistic Organ Dysfunction 2 Score as a continuous variable; Time to CRRT – time in days from intensive care admission to CRRT initiation; CI – Confidence Interval Median (Q1, Q3); n (%);

### Supplemental Table 4. Duration of CRRT based upon severity of ALI and Degree of Fluid Accumulation

PaO2/FiO2 Ratio = Mild

| **Characteristic** | **N** | **≤10%**, N = 37 | **>10-20%**, N = 15 | **>20%**, N = 15 | **p-value** |
| --- | --- | --- | --- | --- | --- |
| **CRRT duration** | 67 | 8 (5, 13) | 5 (4, 12) | 9 (4, 38) | 0.545 |

PaO2/FiO2 Ratio = Moderate

| **Characteristic** | **N** | **≤10%**, N = 81 | **>10-20%**, N = 28 | **>20%**, N = 44 | **p-value** |
| --- | --- | --- | --- | --- | --- |
| **CRRT duration** | 142 | 6 (3, 12) | 7 (3, 17) | 6 (4, 12) | 0.949 |

PaO2/FiO2 Ratio = Severe

| **Characteristic** | **N** | **≤10%**, N = 56 | **>10-20%**, N = 18 | **>20%**, N = 29 | **p-value** |
| --- | --- | --- | --- | --- | --- |
| **CRRT duration** | 103 | 6 (3, 19) | 8 (4, 15) | 6 (4, 12) | 0.87 |

Kruskal-Wallis rank sum test

### Abbreviations: FA^1^ –the percentage fluid accumulation as a continuous variable at the start of CRRT; CRRT – Continuous Renal Replacement Therapy; PF Ratio^2^ – PaO_2_ to FiO_2_ ratio as a continuous variable at the start of CRRT OR – Odds Ratio; MAKE90^3^ – Major Adverse Kidney Events at 90 days after acute kidney injury diagnosis; PELOD-2^4^ – Pediatric Logistic Organ Dysfunction 2 Score as a continuous variable; Time to CRRT – time in days from intensive care admission to CRRT initiation; CI – Confidence Interval Median (Q1, Q3); n (%);

### Supplemental Table 5. Number of IMV free days based upon severity of ALI and Degree of Fluid Accumulation

PaO2/FiO2 Ratio = Mild

| **Characteristic** | **N** | **≤10%**, N = 37 | **>10-20%**, N = 15 | **>20%**, N = 15 | **p-value** |
| --- | --- | --- | --- | --- | --- |
| **28-day ventilation free days** | 67 | 16 (0, 28) | 0 (0, 19) | 6 (0, 28) | 0.51 |

PaO2/FiO2 Ratio = Moderate

| **Characteristic** | **N** | **≤10%**, N = 81 | **>10-20%**, N = 28 | **>20%**, N = 44 | **p-value** |
| --- | --- | --- | --- | --- | --- |
| **28-day ventilation free days** | 142 | 9 (0, 23) | 0 (0, 22) | 13 (0, 28) | 0.56 |

PaO2/FiO2 Ratio = Severe

| **Characteristic** | **N** | **≤10%**, N = 56 | **>10-20%**, N = 18 | **>20%**, N = 29 | **p-value** |
| --- | --- | --- | --- | --- | --- |
| **28-day ventilation free days** | 103 | 0 (0, 16) | 3 (0, 17) | 0 (0, 14) | 0.25 |

Kruskal-Wallis rank sum test

### Abbreviations: FA^1^ –the percentage fluid accumulation as a continuous variable at the start of CRRT; CRRT – Continuous Renal Replacement Therapy; PF Ratio^2^ – PaO_2_ to FiO_2_ ratio as a continuous variable at the start of CRRT OR – Odds Ratio; MAKE90^3^ – Major Adverse Kidney Events at 90 days after acute kidney injury diagnosis; PELOD-2^4^ – Pediatric Logistic Organ Dysfunction 2 Score as a continuous variable; Time to CRRT – time in days from intensive care admission to CRRT initiation; CI – Confidence Interval Median (Q1, Q3); n (%);

### Supplemental Table 6. Rate of Renal recovery at 90 days based upon ALI severity and Degree of Fluid Accumulation

PaO2/FiO2 Ratio = Mild

| **Characteristic** | **N** | **Not recovered**, N = 43 | **Recovered**, N = 24 | **p-value** |
| --- | --- | --- | --- | --- |
| **%FO category** | 67 |  |  | 0.08 |
| ≤10% |  | 27 (63%) | 10 (42%) |  |
| >10-20% |  | 10 (23%) | 5 (21%) |  |
| >20% |  | 6 (14%) | 9 (38%) |  |

PaO2/FiO2 Ratio = Moderate

| **Characteristic** | **N** | **Not recovered**, N = 88 | **Recovered**, N = 54 | **p-value** |
| --- | --- | --- | --- | --- |
| **%FO category** | 144 |  |  | 0.99 |
| ≤10% |  | 45 (50%) | 28 (52%) |  |
| >10-20% |  | 17 (19%) | 10 (19%) |  |
| >20% |  | 26 (30%) | 16 (30%) |  |

PaO2/FiO2 Ratio = Severe

| **Characteristic** | **N** | **Not recovered**, N = 76 | **Recovered**, N = 27 | **p-value** |
| --- | --- | --- | --- | --- |
| **%FO category** | 103 |  |  | 0.25 |
| ≤10% |  | 45 (59%) | 11 (41%) |  |
| >10-20% |  | 12 (16%) | 6 (22%) |  |
| >20% |  | 19 (25%) | 10 (37%) |  |

Fisher’s Exact test

### Abbreviations: FA^1^ –the percentage fluid accumulation as a continuous variable at the start of CRRT; CRRT – Continuous Renal Replacement Therapy; PF Ratio^2^ – PaO_2_ to FiO_2_ ratio as a continuous variable at the start of CRRT OR – Odds Ratio; MAKE90^3^ – Major Adverse Kidney Events at 90 days after acute kidney injury diagnosis; PELOD-2^4^ – Pediatric Logistic Organ Dysfunction 2 Score as a continuous variable; Time to CRRT – time in days from intensive care admission to CRRT initiation; CI – Confidence Interval Median (Q1, Q3); n (%);

### Supplemental Table 7. Number of 28-day IMV free days based upon PF Ratio and number of days to net negative fluid accumulation

PaO2/FiO2 Ratio = Mild

| **Characteristic** | **N** | **≤ Median**  N = 45 | **> Median**  N = 15 | **p-value** |
| --- | --- | --- | --- | --- |
| **28-day ventilator free days** | 60 | 19 (0, 28) | 0 (0, 22) | 0.16 |

PaO2/FiO2 Ratio = Moderate

| **Characteristic** | **N** | **≤ Median**  N = 98 | **> Median**  N = 25 | **p-value** |
| --- | --- | --- | --- | --- |
| **28-day ventilator free days** | 123 | 14 (0, 23) | 13 (0, 28) | 0.62 |

PaO2/FiO2 Ratio = Severe

| **Characteristic** | **N** | **≤ Median**  N = 65 | **> Median**  N = 19 | **p-value** |
| --- | --- | --- | --- | --- |
| **28-day ventilator free days** | 84 | 0 (0, 17) | 0 (0, 8) | 0.17 |

Wilcoxon rank sum test

### Abbreviations: FA^1^ –the percentage fluid accumulation as a continuous variable at the start of CRRT; CRRT – Continuous Renal Replacement Therapy; PF Ratio^2^ – PaO_2_ to FiO_2_ ratio as a continuous variable at the start of CRRT OR – Odds Ratio; MAKE90^3^ – Major Adverse Kidney Events at 90 days after acute kidney injury diagnosis; PELOD-2^4^ – Pediatric Logistic Organ Dysfunction 2 Score as a continuous variable; Time to CRRT – time in days from intensive care admission to CRRT initiation; CI – Confidence Interval Median (Q1, Q3); n (%);

### References.

1. Goldstein SL, Currier H, Graf JM, Cosio CC, Brewer ED, Sachdeva R. Outcome in Children Receiving Continuous Venovenous Hemofiltration. *Pediatrics*. 2001;107(6):1309-1312. doi:10.1542/peds.107.6.1309
2. Force ADT. Acute Respiratory Distress Syndrome: The Berlin Definition. *Jama*. 2012;307(23):2526-2533. doi:10.1001/jama.2012.5669
3. IV FTB, Shaw AD. Clinical Trial Endpoints in Acute Kidney Injury. *Nephron Clin Pract*. 2014;127(1-4):89-93. doi:10.1159/000363725
4. Leteurtre S, Duhamel A, Salleron J, et al. PELOD-2. *Crit Care Med*. 2013;41(7):1761-1773. doi:10.1097/ccm.0b013e31828a2bbd
